# Suicidality in a population of young adults: data from a Consultation-Liaison Psychiatry setting

**DOI:** 10.1192/j.eurpsy.2026.12016

**Published:** 2026-07-31

**Authors:** A. Sciolto, G. Cinesi, C. M. Carioti, F. Di Maio, E. S. Gaias, F. De Giorgi, G. Menculini, A. Tortorella

**Affiliations:** 1 Section of Psychiatry, Department of Medicine and Surgery, University of Perugia; 2 Division of Psychiatry, General Hospital of Perugia, Perugia, Italy

## Abstract

**Introduction:**

Adolescents and young adults exhibit elevated suicide risk, which represents a critical public health issue. This study seeks to delineate the clinical and psychosocial characteristics of youths referred for psychiatric evaluation after a suicide attempt (SA).

**Objectives:**

The primary objective of the present investigation was to describe the sociodemographic, clinical, and psychopathological features of adolescents and young adults evaluated after a SA, compared with peers referred for other psychiatric reasons. By identifying distinctive correlates of suicidality, the study intended to refine early risk assessment and the development of targeted preventive strategies.

**Methods:**

We conducted an observational study with cross-sectional design at the University Hospital of Perugia, Italy, including 128 individuals aged 12 - 35, who underwent a first psychiatric assessment during hospitalization. Participants were categorized into two subgroups: those referred following a suicide attempt and those referred for alternative psychiatric concerns. Moreover, the mean age of the SA group was lower compared to the non-SA group (22.81 ± 6.73 years vs. 25.34 ± 6.87 years, p = 0.038). Data were extracted from standardized consultation reports and encompassed sociodemographic, clinical, and psychopathological variables. Comparative bivariate analyses were performed to examine between-group differences (p < 0.05).

**Results:**

Our sample was predominantly composed of female patients (n = 89; 69.5%), with a mean age of 23.91 ± 6.9 years. Overall, 72 patients (56.3%) were assessed following a SA. The SA group demonstrated significantly higher rates of unemployment (36,2% vs 15,3%, p = 0,024). Moreover, patients in this group were less frequently parents (2.9% vs. 17.9%, p = 0.011) and less often socially isolated (23.1% vs. 73.3%, p = 0.023). A higher prevalence of positive psychiatric family history (47,5% vs 21,7%, p = 0,022), prior suicide attempts (36,1% vs 10,9%, p = 0,002), previous psychiatric hospitalizations (34, 4% vs 9,1%, p = 0,002), previous enrollment in community mental health service (54,2% vs 32,1%, p = 0,021) and pre-admission insomnia (47,1% vs 11,1%, p = 0,020) was found in the SA group. Finally, patients evaluated after a SA showed lower rates of anxiety compared to the control group (40% vs. 16.1%, p = 0.021) and were less frequently affected by chronic medical conditions (32.7% vs. 9.7%, p = 0.003).

**Conclusions:**

Prominent clinical indicators of suicide risk in youth may include unemployment, psychiatric personal and family history and insomnia. Socio-environmental factors appeared to play a relevant role as potential correlates of suicidality in this population. Early and multidimensional risk assessment, coupled with integrative and multidisciplinary interventions within liaison psychiatry, are required to optimize the identification and prevention of suicidality in adolescents and young adults.

**Disclosure of Interest:**

None Declared

